# Tribal differences in hypertension and cholesterol profiles in Aceh, Indonesia

**DOI:** 10.21542/gcsp.2024.22

**Published:** 2024-04-20

**Authors:** Desiana Desiana, Zainal Abidin Muchlisin, Khairi Suhud, Basri A. Gani

**Affiliations:** 1 Graduate School of Faculty of Mathematics and Applied Sciences, Universitas Syiah Kuala, Banda Aceh, 23111, Indonesia; 2 Faculty of Marine and Fisheries, Universitas Syiah Kuala, Banda Aceh, 23111, Indonesia; 3 Department Chemistry, Faculty of Mathematics and Natural Sciences, Universitas Syiah Kuala, Banda Aceh, 23111, Indonesia; 4 Department of Oral Biology, Dentistry faculty, Universitas Syiah Kuala, Banda Aceh, 23111, Indonesia

## Abstract

Background: One of the factors that contributes to coronary heart disease and stroke is high blood pressure, or hypertension. Hypertension is influenced by race and sex. The objective of this study was to assess the hypertensive population in Aceh by tribal community and to examine the relationship between cholesterol history and hypertension.

Methods: This study used incidental sampling as a non-probability sampling method, in which 152 participants were evaluated for the profile of hypertension with a history of cholesterol. Blood pressure was measured using a blood pressure measuring device. HDL, LDL, triglyceride, and total cholesterol levels were measured using LIPID Pro. Data analysis was performed using the Kruskal–Wallis and Mann–Whitney tests with *p* < 0.05.

Results: The study population (*N* = 152) consisted of 81 males (53%) and 71 females (47%) across the ethnicities of Aceh (64:42%), Gayo (19:13%), Alas (33:22%), and Aneuk Jamee (36:24%). In the male group, hypertension was associated with total cholesterol (*r* = 0.03; *p* = 0.78), HDL (*r* = 0.20; *p* = 0.07), and LDL (*r* = 0.21; *p* = 0.07) levels, whereas in the female group, hypertension was primarily correlated with LDL levels (*r* = 0.20; *p* = 0.09).

Conclusion: In general, hypertension in males and females in the four tribes in Aceh is associated with HDL, LDL, and total cholesterol levels.

## Introduction

High blood pressure (hypertension) is increasing every year in both industrial and developing countries, including Indonesia^1^. Data from the Global Burden of Disease in 2000 showed that hypertension was the root cause of 50% of cardiovascular disease. According to the 2007 Basic Health Research, Indonesia’s third-leading cause of death is hypertension^2^. Hypertension is a condition where blood pressure exceeds normal levels and is known as the “silent killer” as it rarely shows symptoms^3^. Long-term high blood pressure or its persistence can result in kidney failure, coronary heart disease, and stroke^4^. Hypertensive disease can be caused by age, excessive sodium intake, dyslipidemia, diabetes mellitus, or stress^5,6^.

Increased blood vessel fat or atherosclerosis can also cause hypertension by causing blood cells and cholesterol-based plaques to build up on the inside walls of arteries^7^. Blood cholesterol is also associated with hypertension^8^. The role of blood cholesterol levels in terms of increasing obesity and the incidence of hypertension is related to the levels of low-density cholesterol, high-density cholesterol and triglycerides^9^.

Triglycerides are a type of fat that is absorbed through the intestines. They are then broken down by enzymes and sent to the liver, where they are metabolized back into LDL^10^. An increase in carbohydrate levels causes an increase in triglyceride levels. Increased triglyceride levels in the blood, along with elevated cholesterol levels, will lead to plaque buildup in blood vessels and blockages in the coronary arteries. Additionally, the plaque hardens and constricts the blood vessels, reducing blood flow and increasing blood pressure. Elevated triglyceride levels cause a decrease in endothelial function, resulting in impaired vasoregulation, increased arterial vascular stiffness, and impaired nitric oxide synthesis. Nitric oxide is a vasodilator that functions as a regulator of blood flow and pressure, preventing platelet aggregation and adhesion^11^. The assessment of hypertension in Acehnese tribes triggered by increased levels of HDL, LDL, triglycerides, and total cholesterol is expected to provide data related to the relationship between the spread of hypertension triggered by blood fat levels in Acehnese tribes.

## Methodology

This study employed accidental sampling as a sampling strategy. Accidental sampling is a method of selecting a sample by selecting respondents who just happen to be present or are readily available somewhere in accordance with the research environment. Patients with hypertension provided quantitative information about themselves including age, ethnicity, sex, HDL titer, LDL, triglycerides, and total cholesterol. Blood serum was examined at the Clinical Pathology Laboratory of RSUZA Banda Aceh.

The participants in this study were hypertensive patients with a history of hyperlipidemia who were sent to Dr. Zainal Abidin Hospital, Banda Aceh, from regional hospitals (districts/municipalities) in the Aceh province (Ras Gayo, Ras Aneuk Jamee and Ras Aceh). There were 152 study participants in total.

### Patient and sample preparation

The patient must fast for 8–12 h prior to sampling as part of the patient preparation process. Sampling was carried out with 3–5 cc of venous blood and inserted in a yellow vacuum tube. Blood samples were then centrifuged at 3000 rpm for 15 min to separate blood serum and plasma. Subsequently, LDL, HDL, triglycerides, and total cholesterol levels were measured using LIPID Pro (Obanikoro, Lagos, Nigeria). Non-hemolyzed and frozen blood serum was filled in A two mL tube, which was then kept at 2−8 °C for 24 h before testing.

### HDL screening

One hundred microliters of blood was pipetted into a cuvette, which was then read using the apparatus. Both male and female subjects should have levels 30–75 mg/dl.

### LDL screening

The LDL screening procedure follows the HDL screening procedure. However, for both male and female, the typical range for LDL is 66–178 mg/dl.

### Triglyceride screening

Triglyceride screening was performed following the same steps. For both male and female, the typical range is 36-165 mg/dl.

### Total cholesterol screening

Total cholesterol was examined using the CHOD-PAP (Cholesterol Oxidase-Peroxidase Amino antipyrine Phenol) method. The examination was carried out after oxidation and hydrolysis of calorimetric indoctrinating enzymes, namely chinonimine, 4-aminoantipyrine, and phenol with hydrogen peroxide, with the help of a deraxide catalyst. Total cholesterol examination was performed using serum samples at a wavelength of 546 nm, cuvette thickness of one cm, and temperature of 37 °C. To measure the absorbance of the prepared samples, blank, and standard using a photometer, the samples were mixed until homogeneous and incubated for 10 min at room temperature. Furthermore, 200 µl of each sample were placed into the blank. After sorting and organizing the samples for examination, the prepared serum was placed in a photometer, and the findings were recorded. Normal total cholesterol values are 140-200 mg/dl, moderate risk is 200–240 mg/dl and high risk is >240 mg/dl.

### Data analysis

Data on hypertension and total cholesterol in Aceh were compared between racial groups using the Kruskal-Wallis test with *p* < 0.005, while the correlation data (between total cholesterol and hypertension) were analyzed using Spearman’s rho correlation ( *r* = 1).

### Ethics approval and consent to participate

We obtained written approval from the ethics committee of Dr Zainal Abidin Hospital with ethics approval: 149/ETIK-RSUDZA/2023.

## Results

Preparing the research participants or patients with hypertension and a history of cholesterol was the first step in the study. Several checks were made including those for age, sex, and ethnicity as shown in the Table 1. The table shows that there were 152 research participants in this study, predominately male, with the Aceh tribe having the highest representation of males (38 people). The age of the subjects varied from 25 to over 60, but the subjects of this study were dominated by the age group 46-60 years (45 people).

**Table 1:** Patients’ population in Aceh with hypertension and a history of cholesterol by age and tribe.

No	Research participants	Population distribution (Tribe)				Age			Total
		Aceh	Gayo	Alas	Aneuk Jamee	25–45	46–60	>60	
1	Male	38	10	14	19	12	45	24	81
2	Female	26	9	19	17	12	36	23	71

A blood pressure examination was carried out on each subject, with the results listed in Table 2. There were 28 women with normal systole and 32 women with normal diastole. The males had 23 normal systoles and 38 normal diastoles. This table also shows that in the HDL examination, the female group had a higher percentage of risk than the male group (25% for men and 32% for women).

**Table 2:** The results of the analysis of the research participants.

No	Examination type	Research participants	
		Male	Female
1	**High Blood Pressure**		
	Systole		
	100–150 (Normal)	23	28
	151–200 (Moderate risk)	50	31
	>200 (High risk)	8	12
	Diastole		
	<60 (Abnormal)	1	2
	60–90 (Normal)	38	32
	91–100 (Moderate risk)	23	24
	>100 (High risk)	19	13
2	**HDL Level (mg/dl)**		
	<30 (Risk)	20	23
	30–75 (Normal)	61	48
3	**LDL Level (mg/dl)**		
	66–178 (Normal)	22	15
	>178 (High risk)	59	56
4	**Triglyceride Content (mg/dl)**		
	36–165 (Normal)	15	13
	>165 (High risk)	56	58
5	**Total Cholesterol Level (mg/dl)**		
	100–200 (Normal)	15	27
	201–240 (Moderate risk)	38	29
	>240 (High risk)	28	15

**Notes.**

* Correlation significant at 0.05 (2-tailed).

** Correlation significant at 0.01 (2-tailed).

In contrast, high-risk LDL values in the male group were marginally higher (73%) than corresponding values in the female group (69%). However, the number of patients with high-risk triglyceride levels was 82% for women and 69% for males, and male high-risk cholesterol levels reached 35%, with a maximum of 432 mg/dl and a minimum of 241 mg/dl, while women’s levels reached 0.21, with a maximum of 506 mg/dl and a minimum of 242 mg/dl.

The distribution of high blood pressure in male subjects in tribes in Aceh with a history of cholesterol and the correlation of the prevalence of hypertension in male subjects in tribes in Aceh are shown in Tables 3 and 4. According to Table 3, the blood pressure distribution in the group of males with a history of cholesterol can be broken down into the following risk states: systole has a high risk, diastole has a moderate risk, normal HDL, LDL has a high risk, triglycerides and total cholesterol have a high risk.

**Table 3:** Distribution of high blood pressure of male participants with history of cholesterol in tribes in Aceh.

Data description	Hypertension (mmHg)		Cholesterol (mg/dL)				**p-value
	Systole (*n* = 71)	Diastole (*n* = 71)	HDL (*n* = 71)	LDL (*n* = 71)	Triglyceride (*n* = 71)	Cholesterol total (*n* = 71)	
Mean	167,36	96,01	33,74	209,73	219,91	241,94	0
SDV	26,18	20,17	9,42	51,09	81,52	56,84	
Min	120	44	20	69	80	150	
Max	260	191	56	320	600	432	
*p-value	0,26	0,28	**0,01**	0,29	0,19	**0,01**	

**Notes.**

* Kruskal Wallis Test.

** Friedman Test; Mean values are representative of Tribal groups (Aceh 47%; Gayo 12%; Alas 17%; Aneuk Jamee 23%).

**Table 4:** Spearman’s rho variable correlation of incidence of hypertension in male participants with history of cholesterol in tribes in Aceh.

**Correlation variable**	**Spearman’s rho**	**Systole**	**Diastole**	**HDL**	**LDL**	**Triglyceride**	**Cholesterol total**
Tribes	Correlation Coefficient	−0,04	−0,07	−0,22^*^	0,15	0,11	0,29^**^
	Sig. (2-tailed)	0,72	0,53	0,05	0,18	0,31	0,1
	*N*	81	81	81	81	81	81
Systole	Correlation Coefficient		0,69^**^	0,2	0,21	−0,02	0,03
	Sig. (2-tailed)		0	0,07	0,07	0,82	0,78
	*N*		81	81	81	81	81
Diastole	Correlation Coefficient			0,03	−0,08	−0,16	−0,1
	Sig. (2-tailed)			0,79	0,45	0,16	0,38
	*N*			81	81	81	81
HDL	Correlation Coefficient				−0,23^*^	0,02	−0,03
	Sig. (2-tailed)				0,04	0,87	0,81
	*N*				81	81	81
LDL	Correlation Coefficient					−0,01	−0,13
	Sig. (2-tailed)					0,92	0,25
	*N*					81	81
Triglyceride	Correlation Coefficient						0,13
	Sig. (2-tailed)						0,23
	*N*						81

**Notes.**

* Correlation significant at 0.05 (2-tailed).

** Correlation significant at 0.01 (2-tailed).

Tables 5 and 6 present information on the distribution of high blood pressure among female subjects with a history of cholesterol in Aceh tribes as well as the link between the incidence of hypertension among female subjects with a history of cholesterol in Aceh tribes.

**Table 5:** Distribution of high blood pressure of female participants with history of cholesterol in tribes in Aceh.

Data description	Hypertension (mmHg)		Cholesterol mg/dL)				^**^p-value
	Systole (*n* = 71)	Diastole (*n* = 71)	HDL (*n* = 71)	LDL (*n* = 71)	Triglyceride (*n* = 71)	cholesterol total (*n* = 71)	
Mean	163,32	93,14	35,48	218,01	223,93	224,15	0
SDV	31,97	17,11	9,93	71,72	75,55	56,08	
Min	100	46	21	80	76	132	
Max	270	159	57	600	452	506	
*p-value	0,45	0,67	0,27	0,29	0,09	**0,05**	

**Notes.**

* Kruskal Wallis Test.

** Friedman Test; Mean values are representative of Tribal groups (Aceh 37%; Gayo 13%; Alas 27%; Aneuk Jamee 24%).

**Table 6:** Spearman’s rho variable correlation of incidence of hypertension in female participants with history of cholesterol in tribes in Aceh.

**Correlation variable**	**Spearman’s rho**	**Systole**	**Diastole**	**HDL**	**LDL**	**Triglyceride**	**Cholesterol total**
Tribes	Correlation Coefficient	0,15	0,14	−0,06	0,20	−0,19	−0,14
	Sig. (2-tailed)	0,21	0,25	0,63	0,09	0,11	0,24
	*N*	71	71	71	71	71	71
Systole	Correlation Coefficient		0,66^**^	−0,03	0,15	−0,03	−0,19
	Sig. (2-tailed)		0,00	0,80	0,20	0,81	0,12
	*N*		71	71	71	71	71
Diastole	Correlation Coefficient			−0,09	0,20	−0,05	−0,20
	Sig. (2-tailed)			0,48	0,09	0,69	0,10
	*N*			71	71	71	71
HDL	Correlation Coefficient				−0,21	−0,05	−0,08
	Sig. (2-tailed)				0,08	0,66	0,50
	*N*				71	71	71
LDL	Correlation Coefficient					−0,09	0,03
	Sig. (2-tailed)					0,48	0,78
	*N*					71	71
Triglyceride	Correlation Coefficient						0,20
	Sig. (2-tailed)						0,09
	*N*						71

**Notes.**

* Correlation significant at 0.05 (2-tailed).

** Correlation significant at 0.01 (2-tailed).

Men are 2.33 times more likely to have elevated systolic blood pressure than women, while women are more likely to have hypertension after the age of 65 or on reaching menopause, due to hormonal reasons. According to this study’s findings, the male group has higher levels of systole and diastole than the female group as shown in Tables 7 and 8.

**Table 7:** Distribution of high blood pressure of participants with cholesterol history by gender.

Group of participant	*n* = 152					
	Systole (mmHg)	Diastole (mmHg)	HDL (mg/dL)	LDL (mg/dL)	Triglyceride (mg/dL)	Cholesterol total (mg/dL)
	Mean ± SDV	Mean ± SDV	Mean ± SDV	Mean ± SDV	Mean ± SDV	Mean ± SDV
Male	167,35 ± 26,18	96,01 ± 20,17	33,74 ± 9,42	209,73 ± 51,09	219,91 ± 81,52	241,94 ± 56,84
Female	163,32 ± 31,97	93,14 ± 17,11	35,48 ± 9,93	218.01 ± 71,72	223,93 ± 75,55	224,15 ± 56,08
*p-value	0,15	0,52	0,28	0,89	0,74	0,04

**Notes.**

* Mann-Whitney; Male (81.53%), Female (71.47%).

**Table 8:** Spearman’s rho variable correlation of participant’s hypertension incidence with cholesterol history by gender.

**Correlation variable**	**Spearman’s rho**	**Systole**	**Diastole**	**HDL**	**LDL**	**Triglyceride**	**Cholesterol total**
Gender	Correlation Coefficient	−0,12	−0,05	0,08	0,01	0,03	−0,16^*^
	Sig. (2-tailed)	0,15	0,52	0,28	0,90	0,74	0,04
	*N*	152	152	152	152	152	152
Systole	Correlation Coefficient		0.67^**^	0,09	0,05	−0,03	−0,06
	Sig. (2-tailed)		0,00	0,23	0,56	0,76	0,44
	*N*		152	152	152	152	152
Diastole	Correlation Coefficient			−0,02	0,06	−0,11	−0,15
	Sig. (2-tailed)			0,82	0,5	0,19	0,06
	*N*			152	152	152	152
HDL	Correlation Coefficient				−0.21^**^	−0.01	−0.05
	Sig. (2-tailed)				0.01	0.91	0.53
	*N*				152	152	152
LDL	Correlation Coefficient					−0.04	−0.07
	Sig. (2-tailed)					0.62	0.37
	*N*					152	152
Triglyceride	Correlation Coefficient						0.17^*^
	Sig. (2-tailed)						0.04
	*N*						152

**Notes.**

* Correlation significant at 0.05 (2-tailed).

** Correlation significant at 0.01 (2-tailed).

## Discussion

This study found that race (ethnicity) had no effect on high blood pressure but only on total cholesterol, indicating that nutrition may play a major role in the rise in cholesterol levels. Genes that cause hypertension are particularly affected by race. Lackland showed that the population attributable risk for hypertension and 30-year mortality among white men was 23.8% compared with 45.2% among black men and 18.3% for white women compared with 39.5% for black women^21^. This difference can be identified as a genotypic factor in each race that is susceptible to the genes that cause hypertension^12^. In addition, Brondolo et al. reported that racism or discrimination leads to a relationship between stress exposure and reactivity, as well as the relationship between risk factors for hypertension and their influences, including food, nutrition, and lifestyle variations^13^.

The Indonesian Ministry of Health (Rikesda) reported in 2022 that hypertension affects people between the ages of 31 and 44 (31.6%), 45 and 54 (45.3%), and 55 and 64 (55.2%), respectively. According to the results of this study, age 46–60 years was associated with the most prevalent incidence of hypertension in both the male (56%) and female (51%) categories.

Peltzer (2013) reported that the prevalence of hypertension in the population was highest among men 77.3% (with women at 25.6%)^14^. The risk of cardiovascular disease is increased in older persons (50+ years) due to the high prevalence of hypertension. Odden et al. reported that high blood pressure is a serious health issue that is prevalent among older persons. Age-related changes in blood vessel activity have an impact on this. As arteries stiffen, blood pressure increases^15^.

In general, blood pressure distribution in the male group with a history of cholesterol can be divided into the following categories: systole has a high risk, diastole has a moderate risk, HDL is normal, LDL is high risk, triglycerides and total cholesterol have a high risk.

In the female group, systole was at high risk, diastole was at moderate risk, HDL was normal, LDL and triglycerides were at high risk, and total cholesterol was at moderate risk.

Due to the relationship between high blood pressure and high cholesterol, whereby cholesterol and calcium plaques cause the arteries to become hard and narrow, which makes the heart work harder to pump blood, high blood pressure is connected with elevated LDL levels^16^.

In this study, since HDL levels were normal, the research participants’ overall ability to lower hypertension by transferring cholesterol to prevent plaque accumulation that would restrict blood flow and possibly obstruct blood arteries was present. High levels of HDL can lower the risk of hypertension, heart disease, and stroke, whereas low levels of HDL are linked to worsening kidney function in non-diabetics. In older persons, hypertension and low HDL cholesterol are associated with worsening renal function^17^.

Triglycerides and blood pressure are favourably correlated (both systolic and diastolic)^18^. In contrast, in this study it was reported that in both male and female groups there was no correlations of hypertension with triglycerides. Obesity and metabolic syndrome are two conditions that can be increased by elevated triglycerides, which can also result in an increase in fat that raises blood pressure^19^.

Pathophysiologically, cholesterol-induced hypertension occurs when the body is unable to clear cholesterol from the bloodstream and excess cholesterol deposits along the artery walls. The arteries become stiff and narrowed owing to the deposits, and the heart has to work overtime to pump blood through them, leading to an increase in blood pressure^20^.

## Conclusion

The prevalence of high blood pressure is influenced by age, with older individuals having a higher risk of high blood pressure. However, older adult women are more likely to have higher BP than older adult men. Additionally, the levels of LDL and total cholesterol are linked to an increase in high blood pressure. Triglyceride levels have a positive relationship with high blood pressure, but in this study, triglyceride levels did not show any significant correaltion.

## Author statements

Conception: Desiana, Zainal Abidin Muchlisin.

Design: Khairi Suhud, Basri A. Gani.

Supervision: Desiana, Basri A. Gani

Funding: Desiana

Materials: Zainal Abidin Muchlisin, Khairi Suhud

Data Collection and/or Processing: Desiana

Analysis and/or Interpretation: Desiana, Khairi Suhud, Basri A. Gani

Literature Review: Desiana, Khairi Suhud

Writing: Desiana

Critical Review: Zainal Abidin Muchlisin, Basri A. Gani

## Competing Interest

The authors declare that they have no competing interest.

## Acknowledgement

The author thanks to the promoters from Universitas Syiah Kuala for all their knowledge and support in this process and all participants who have supported and provided information for this study.

## Abbreviations

HDL: High Density Lipoprotein
LDL: Low Density Lipoprotein
CHOD-PAP: Cholesterol Oxidase-Peroxidase Amino Antipyrine Phenol

## References

[1] Peltzer K, Pengpid S (2018). The prevalence and social determinants of hypertension among adults in Indonesia: A cross-sectional population-based national survey. International Journal of Hypertension.

[2] Hussain MA, Al Mamun A, Peters SA, Woodwar M, Huxley RR (2016). The burden of cardiovascular disease attributable to major modifiable risk factors in Indonesia. J Epidemiol.

[3] WHO A Global Brief on Hypertension: Silent Killer, Global Public Health Crisis. 2013. https://www.who.int/publications/i/item/a-global-brief-on-hypertension-silent-killer-global-public-health-crisis-world-health-day-2013.

[4] Wajngarten M, Silva GS (2019). Hypertension and stroke: update on treatment. Eur Cardiol.

[5] Otsuka T, Takada H, Nishiyama Y, Kodani E, Saiki Y, Kato K, Kawada T (2016). Dyslipidemia and the risk of developing hypertension in a working-age male population. J Am Heart Assoc.

[6] Wu Y, Ding Y, Tanaka Y, Zhang W (2014). Risk factors contributing to type 2 diabetes and recent advances in the treatment and prevention. Int J Med Sci.

[7] Rafieian-Kopaei M, Setorki M, Doudi M, Baradaran A, Nasri H (2014). Atherosclerosis: Process, indicators, risk factors and new hopes. Int J Prev Med.

[8] Ariyanti R, Besral B (2019). Dyslipidemia associated with hypertension increases the risks for coronary heart disease: A case-control study in harapan kita hospital, national cardiovascular center, Jakarta. J Lipids.

[9] Baragetti A, Norata G, Sarcina C, Rastelli F, Grigore L, Garlaschelli K, Catapano AL (2013). High density lipoprotein cholesterol levels are an independent predictor of the progression of chronic kidney disease. Journal of Internal Medicine.

[10] Lamm S (2015). Fighting Fat: Break the Dieting Cycle and get Healthy for Life!.

[11] Förstermann U, Sessa WC (2012). Nitric oxide synthases: Regulation and function. Eur Heart J.

[12] Zilbermint M, Hannah-Shmouni F, Stratakis CA (2019). Genetics of hypertension in African Americans and others of African descent. Int J Mol Sci.

[13] Brondolo E, Love EE, Pencille M, Schoenthaler A, Ogedegbe G (2011). Racism and hypertension: A review of the empirical evidence and implications for clinical practice. Am J Hypertens.

[14] Peltzer K, Phaswana-Mafuya N (2013). Hypertension and associated factors in older adults in South Africa. Cardiovasc J Afr.

[15] Odden MC, Beilby PR, Peralta CA (2015). Blood pressure in older adults: the importance of frailty. Curr Hypertens Rep.

[16] Solymar M, Ivic I, Poto L, Hegyi P, Garami A, Hartmann P, Gyöngyi Z (2018). Metformin induces significant reduction of body weight, total cholesterol and LDL levels in the elderly–a meta-analysis. PLOS ONE.

[17] Odden MC, Tager IB, Gansevoort RT, Bakker SJ, Fried LF, Newman AB, Shlipak MG (2013). Hypertension and low HDL cholesterol were associated with reduced kidney function across the age spectrum: A collaborative study. Ann Epidemiol.

[18] da Silva A, Caldas APS, Hermsdorff HHM, Bersch-Ferreira ÂC, Torreglosa CR, Weber B, Bressan J (2019). Triglyceride-glucose index is associated with symptomatic coronary artery disease in patients in secondary care. Cardiovasc Diabetol.

[19] Nordestgaard BG (2016). Triglyceride-rich lipoproteins and atherosclerotic cardiovascular disease: new insights from epidemiology, genetics, and biology. Circ Res.

[20] Rodriguez-Mateos A, Ishisaka A, Mawatari K, Vidal-Diez A, Spencer JP, Terao J (2013). Blueberry intervention improves vascular reactivity and lowers blood pressure in high-fat-high-cholesterol-fed rats. Br J Nutr.

[21] Lackland DT (2014). Racial differences in hypertension: implications for high blood pressure management. Am J Med Sci.

